# Critical role of CDK11^p58^ in human breast cancer growth and angiogenesis

**DOI:** 10.1186/s12885-015-1698-7

**Published:** 2015-10-15

**Authors:** Yayun Chi, Sheng Huang, Haojie Peng, Mengying Liu, Jun Zhao, Zhiming Shao, Jiong Wu

**Affiliations:** 1 Department of Breast Surgery, Breast Cancer Institute, Fudan University Shanghai Cancer Center, Shanghai, 200032 China; 2 School of Biomedical Engineering, hanghai Jiao Tong University, Shanghai, 200240 China

**Keywords:** CDK11^p58^, Angiogenesis, Kinase activity, VEGF

## Abstract

**Background:**

A capillary network is needed in cancer growth and metastasis. Induction of angiogenesis represents one of the major hallmarks of cancer. CDK11^p58^, a Ser/Thr kinase that belongs to the Cell Division Cycle 2-like 1 (CDC2L1) subfamily is associated with cell cycle progression, tumorigenesis, sister chromatid cohesion and apoptotic signaling. However, its role in breast cancer proliferation and angiogenesis remains unclear.

**Methods:**

Tumorigenicity assays and blood vessel assessment in athymic mice were used to assess the function of CDK11^p58^ in tumor proliferation and angiogenesis. CCK-8 assay was used to detect breast cancer cell growth. Immunohistochemistry was used to detect the expression of vascular endothelial growth factor (VEGF), CD31 and CD34 in CDK11 positive patient breast cancer tissues. Dual-Luciferase array was used to analyze the function of CDK11^p58^ in the regulation of VEGF promoter activity. Western blot was used to detect related protein expression levels.

**Results:**

CDK11^p58^ inhibited breast cancer growth and angiogenesis in breast cancer cells and in nude mice transplanted with tumors. Immunohistochemistry confirmed that CDK11^p58^ was negatively associated with angiogenesis-related proteins such as VEGF, CD31 and CD34 in breast cancer patients. Real-time PCR and dual-luciferase assay showed CDK11^p58^ inhibited the mRNA levels of VEGF and the promoter activity of VEGF. As CDK11^p58^ is a Ser/Thr kinase, the kinase-dead mutant failed to inhibit VEGF mRNA and promoter activity. Western blot analysis showed the same pattern of related protein expression. The data suggested angiogenesis inhibition was dependent on CDK11^p58^ kinase activity.

**Conclusion:**

This study indicates that CDK11^p58^ inhibits the growth and angiogenesis of breast cancer dependent on its kinase activity.

**Electronic supplementary material:**

The online version of this article (doi:10.1186/s12885-015-1698-7) contains supplementary material, which is available to authorized users.

## Background

Blood vessels deliver oxygen and nutrients to every part of the body, but also nourish diseases such as cancer [1]. A capillary network from the surrounding host tissue is needed both in cancer proliferation and in cancer metastasis [2]. Angiogenesis is a physiological multi-step process that includes endothelial cell growth and movement [3]. Induction of angiogenesis represents one of the major hallmarks of cancer [4], and plays important roles in wound healing, endothelial cell-mediated degradation of the extracellular matrix, and the transition of benign tissues into solid tumors [5]. Therefore, there is a great and urgent need to study the regulation and elucidate the mechanisms of cancer angiogenesis. Vascular endothelial growth factor (VEGF) is a predominant activator of endothelial cell functions such as new blood vessel formation (angiogenesis) during development [6]. Through a VEGF-induced signaling pathway, VEGF plays a vital role in the proliferation, migration, and invasion of vascular endothelial cells. In addition, other growth factors such as integrins, matrix metalloproteinases (MMPs) and growth factor receptors (GFRs) also stimulate angiogenesis [1]. As previously reported, VEGF is an important angiogenic factor in human breast cancer [7]. Microvessel density in areas of intense neovascularization in invasive breast carcinoma is an independent and highly significant prognostic indicator for overall and relapse-free survival in patients with early-stage breast carcinoma [8].

CDK11^p58^, a G2/M phase protein associated with cell cycle progression and tumorigenesis [9], is a centrosome-associated mitotic kinase involved in centrosome maturation and bipolar spindle formation and is required for centriole duplication and Plk4 recruitment to mitotic centrosomes [10, 11]. Previously, we found that CDK11^p58^ inhibited the proliferation of prostate cancer and was involved in regulation of androgen and estrogen signaling [12–14]. In addition, our previous study demonstrated that CDK11^p58^ inhibited ERα-positive breast cancer invasion by targeting integrin β3 via the repression of ERα signaling [15] and also we found breast cancers transfected with CDK11^p58^ grew slowly compared with the control cell lines, so we speculated that CDK11^p58^ might inhibit the growth of breast cancer.

In the current study, we evaluated the direct anti-tumor and anti-angiogenic effects of CDK11^p58^ in breast cancer. An *in vivo* model of human breast cancer cell xenografts in nude mice was used to assess the effects and mechanism of CDK11^p58^ on tumor growth and angiogenesis. We sought to determine the potential role of CDK11^p58^ in breast cancer growth and angiogenesis as well as the underlying mechanisms.

## Methods

### Samples

A tissue array including 32 breast cancer patient cancerous tissues were obtained from the tissue bank of Fudan University Shanghai Cancer Center in 2010. This study was approved by the Ethical Committee of our Cancer Center and written informed consent was obtained from each patient.

### Materials

Fetal bovine serum (FBS), Dulbecco’s modified Eagle medium (DMEM), 1640 and expression vector pcDNA3.0 were purchased from Invitrogen (Invitrogen, USA). Mouse and rabbit secondary antibodies for immunohistochemistry (IHC) were purchased from Cell Signaling (CST, USA). Anti-HA and anti-CDK11 polyclonal antibodies were purchased from Santa Cruz Biotechnology (Dallas, Texas, USA). VEGF, CD31, CD34, integrin β3, mmp3 and mmp9 were all purchased from Epitomics Company (Abcam, Cambridge, MA USA). Anti-GAPDH antibodies was purchased from Proteintech (Beijing, China). A dual luciferase reporter assay system was purchased from Promega (Beijing, China).

### Cell culture and cell transfections

293 T, MCF7, MDA-MB-231 and T47D cell lines were obtained from our laboratory cell bank. 293 T, MCF-7 and T47D cells were grown using DMEM supplemented with 10 % FBS, 100 μg/ml penicillin, and 100 μg/ml streptomycin (Cat. 10378–016, Invitrogen) at 37 °C and 5 % CO_2_. MDA-MB-231 cells were cultured using F15 supplemented with 10 % FBS, 100 μg/ml penicillin and 100 μg/ml streptomycin at 37 °C and 5 % CO_2_. Transient transfection for luciferase assays was performed using 96-well plates (1 × 10^4^ cells per well) with 200 ng of total plasmids and Lipofectamine 2000 reagent (Cat.11668-019, Invitrogen) according to the manufacturer’s instructions.

### Stable expression of CDK11^p58^ with retroviral vector

Human CDK11^p58^ was cloned into pBabe-puro vector for ectopic expression of CDK11^p58^. MDA-MB231 and T47D cells were infected with pBabe-puro vector control or CDK11^p58^-overexpression virus and selected by Puromycin. The expression levels of CDK11^p58^ in MDA-MB231 and T47D were confirmed by Western blot assay.

### Tumorigenicity assays and blood vessel assessment in athymic mice

Female athymic BALB/c *nu*/*nu* mice, 4–6 weeks old, were obtained from the Shanghai Institute of Materia Medica, Chinese Academy of Sciences. All studies on mice were conducted in accordance with the National Institute of Health (NIH) ‘Guide for the Care and Use of Laboratory Animals’. The study protocol was approved by the Shanghai Medical Experimental Animal Care Committee. Animals were divided into four groups: MDA-MB-231/vector and MDA-MB-231/CDK11^p58^, T47D/vector and T47D/CDK11^p58^. Each group contained 16 mice. Cells (MDA-MB-231, 1.5 × 10^6^ and T47D, 1 × 10^7^) were injected into the No.4 pairs of mammary fat pad of mice. Animals were monitored every 2 days for tumor growth and general health. Tumor sizes were measured with caliper and calculated by the formula V = (W) ^2^xL/2. Animals were sacrificed and autopsied at 6 weeks after cell inoculation. To confirm the expression of the indicated proteins, sections were cut at 50 μm intervals and stained with hematoxylin and eosin (H&E) and by IHC.

For blood vessels imaging preparation, the image contrast agent, barium sulfate suspended in glycerol (50 % water solution; a concentration of 0.5 mg/mL), was injected into the deeply anesthetized mouse ascending aorta. Then the tumors were excised and fixed by 4 % paraformaldehyde followed by graded ethanol. The microangiography for blood vessels was performed at the Beamline BL13W1, the biomedical application station of the Shanghai Synchrotron Radiation Facility (SSRF) in China. The maximum light size of the beam was 45 mm (horizontal) × 5 mm (vertical) at the object position at 16 keV. Projections of tumor samples in nude mice were then recorded using SSRF. The slice images were reconstructed using the filtered back projection (FBP) algorithm. The vessels of tumor were segmented from these slice images after reducing noise by using Gauss smoothing filter in Matlab. Moreover, thinning algorithm was applied to extract the skeletons of vessels in order to evaluate the status of tumor. After these image pre-processing, micro vessel density (MVD), number of vessel branches and number of vessel nodes were computed in each tumor sample.

### Cell counting kit-8 assay

Stable transfected cells were seeded in a 96-well plate at 5 × 10^3^ cells per well and then cultured for 4 days. A volume of 10 μl of CCK-8 (Cell Counting Kit-8, C0038, Beyotime, Shanghai, China) solution was added to each well of the plate and incubated at 37 °C for 4 h. The absorbance at 450 nm was measured to represent the cell viability.

### Immunohistochemistry

Expression levels of CDK11 (Sc-928, Santa Cruz, USA), VEGF (ab46154, Abcam, USA), CD31 (GM082329, GeneTech, Shanghai, China), and CD34 (GM716529, GeneTech) in postoperative paraffin-embedded tumor specimens from breast cancer patients and mice tumor tissues were detected with IHC. The concentrations of antibodies used are as follows: CDK11, 1:100; VEGF, 1:100; CD31, 1:50; and CD34, 1:50. The Envision and diaminobenzidine (DAB) Color Kit was purchased from Gene Tech Company Limited (Shanghai, China). The staining procedures strictly followed the supplier’s recommendation. The staining index (SI, range 0–9) was considered as the product of the intensity score (0, no staining; 1+, faint/equivocal; 2+, moderate; 3+, strong) and the distribution score (0, no staining; 1+, staining of <10 % of cells; 2+, between 10 % and 50 % of cells; and 3+, >50 % of cells). For CDK11 protein in this study, a moderate/strong staining (SI = 3–9) was defined as positive or high staining, and a weak or negative staining (SI = 0–2) was defined as negative or low staining.

### *In vitro* angiogenesis model

Human Umbilical Vein Endothelial Cells (HUVEC), which were obtained from our laboratory cell bank were suspended in culture medium from stable cell lines and then plated onto a thin layer (300 ml) of basement membrane matrix (Matrigel; BD Biosciences) in 24-well plates at 1 × 10^4^ cells/well. After 12 h, the medium was removed, cells were fixed, and images of cells were obtained with a light microscope (Laica) at × 20 magnification. Quantification of the tubular structures (anastomosing tubules) was performed by counting the number of complete circles produced by interlinking tubular HUVECs [16].

### Dual luciferase reporter assays

293 T, T47D and MCF-7 cells were cotransfected with a VEGF promoter luciferase reporter construct (100 ng) [17], a control Renilla luciferase plasmid (pRL) (1 ng), CDK11^p58^ or other mutants. Total plasmid DNA was adjusted to 300 ng with an empty pcDNA vector. At 48 h post-transfection, a dual luciferase reporter gene assay (Promega) was performed following the instructions using a SynergyHT Multi-Mode Microplate Reader (BioTek, USA).

### Western blot analysis

Cell pellets were lysed, protein extracts were quantitated, loaded onto a 10 % sodium dodecyl sulfate–polyacrylamide gel, electrophoresed, and transferred to a nitrocellulose membrane. The membrane was incubated with primary antibody, washed, and incubated with horseradish peroxidase (HRP)–conjugated secondary antibody (Cell Signaling). Detection was performed usig chemiluminescent Western detection kit. GAPDH was using as a loading control. The quantification of immunoblotting was done by the Photoshop Software.

### Statistical analysis

Results are either representative or are the mean of at least three independent experiments performed in triplicate. Statistical analysis was performed using ANOVA test and Student’s t-test for unpaired data (Prism, GraphPad). Chi-squared test analyses were performed using SPSS (version 19.0; SPSS Company). *P* < 0.05 was considered statistically significant.

## Results

### CDK11^p58^ inhibits the growth of breast cancer

**Fig. 1:**
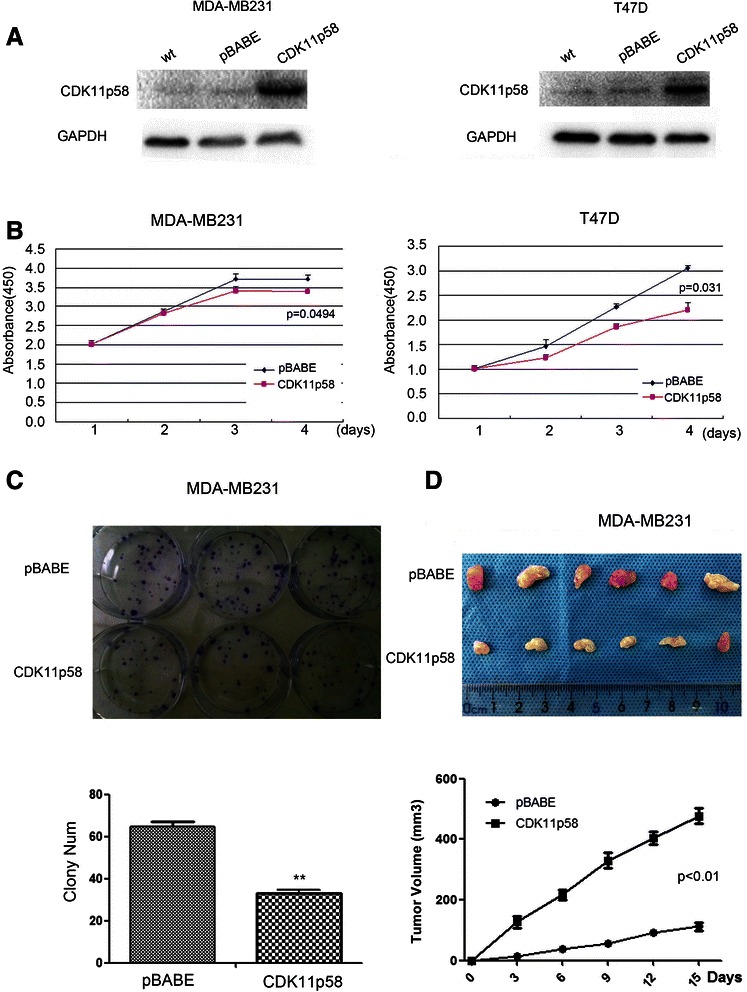
CDK11^p58^ inhibits the proliferation of breast cancer. (**a**) CDK11^p58^ expression was detected by western blot assay in a CDK11^p58^ stable cell line in MDA-MB231 and T47D cells. (**b**) CCK-8 proliferation analysis of CDK11^p58^ stable transfected breast cancer cells MDA-MB231 and T47D compared with controls. (**c**) Colony formation of human breast cancer cells stably transfected with CDK11^p58^ or pcDNA3.0. **P < 0.01. (**d**) Tumorigenesis after injection of MDA-MB231 cells stably expressing CDK11^p58^ or control pBABE. Growth curve with CDK11^p58^ stable expression and controls was also shown

To evaluate the role of CDK11^p58^ in breast cancer, we first constructed CDK11^p58^ stable breast cancer cell lines in ER negative MDA-MB231 and ER positive T47D. Western blot assay showed that CDK11^p58^ was more highly expressed in the two stable cell lines than the control pBABE group and wild type group (Fig. 1a). By Cell Counting Kit-8 assay, we found that CDK11^p58^ inhibited breast cancer cell gowth compared with the pBABE control both in MDA-MB231 and T47D cells (Fig. 1b). Colony formation assay was used to examine the effect of CDK11^p58^ in tumorigenesis and also demonstrated that CDK11^p58^ inhibited the growth and tumorigenesis of breast cancer cells (Fig. 1c, Additional file 1: Figure S1A).

Then we further investigated the role of CDK11^p58^ in tumor growth by using an *in vivo* orthotopic xenograft tumor model in athymic mice. MDA-MB-231/vector/CDK11^p58^ or T47D/vector/CDK11^p58^ cells were injected into the No. 4 mammary fat pad of athymic mice. At 6 weeks, we measured the size of tumors and monitored tumor cell growth. CDK11^p58^ inhibited *in vivo* tumor growth significantly (Fig. 1d, Additional file 1: Figure S1B).

### CDK11^p58^ inhibits the angiogenesis of breast cancer

**Fig. 2:**
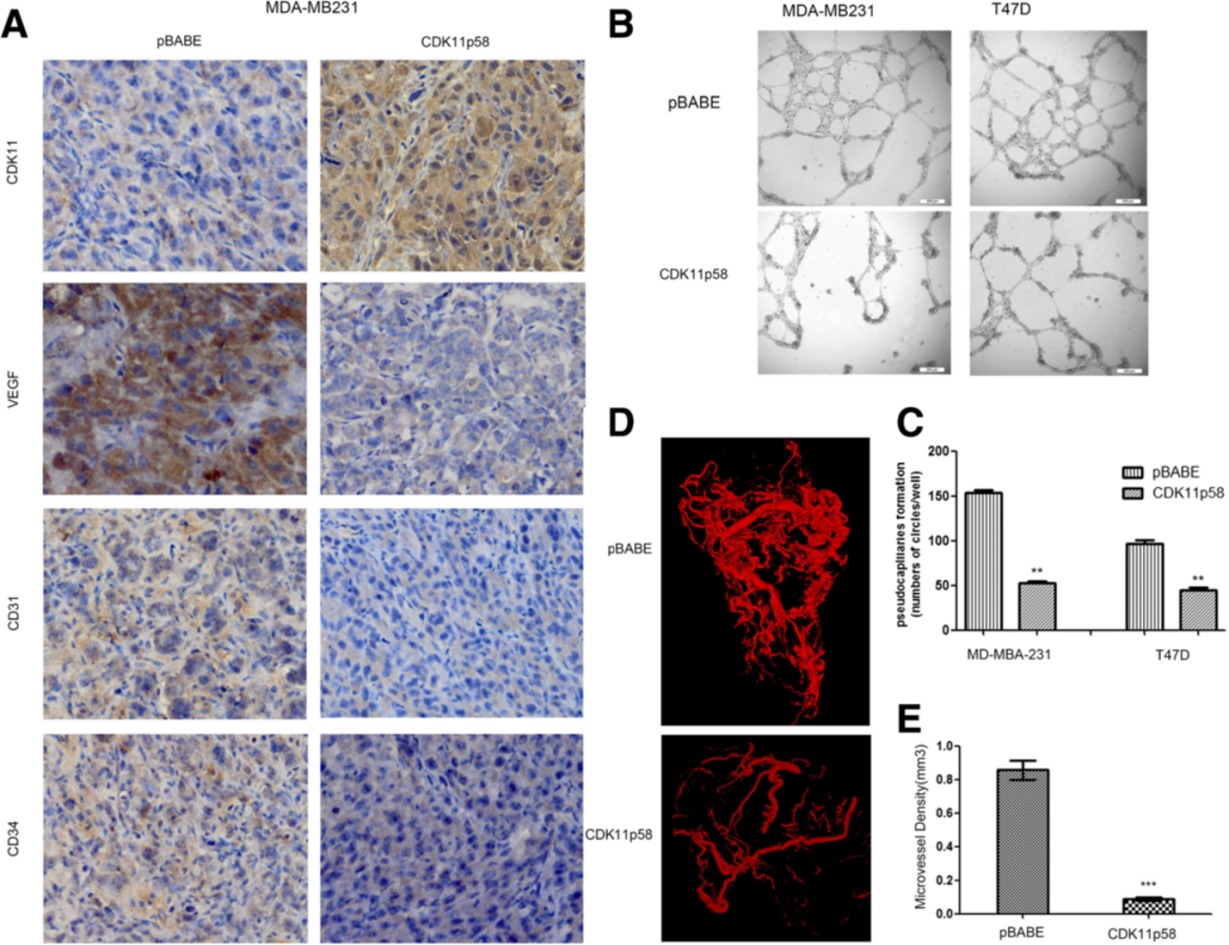
CDK11^p58^ inhibits the angiogenesis of breast cancer. (**a**) Association of CDK11^p58^ expression and VEGF expression in breast cancer in nude mice. Immunohistochemical staining for the expression of CDK11, VEGF, CD31, CD34 in human breast cancer tissues. (**b**) Representative pictures of pseudocapillary formation in matrigel from HUVECs in 0.1 % FBS exposed to breast cancer cell culture at 12 h after cell seeding. (**c**) Quantification of pseudocapillaries obtained by counting numbers of complete circles/wells. Numbers represent the mean of 6 samples ± SEM of three experiments run in triplicate. (**d**) CDK11^p58^ inhibits the vascularization of MDA-MB231 xenograft tumors in mice. The images were reconstructed using the filtered back projection (FBP) algorithm. (**e**) Quantitative analysis of angiogenesis of MDA-MB231 xenograft tumors in implants. For each condition (*n* = 6), the means of 6 samples ± SD are shown. **P < 0.01, CDK11^p58^ group compared to the pBABE group

In the nude mice tumor model, we detected the cancer tissue expressions of CDK11^p58^, VEGF, CD31 and CD34 by IHC. CDK11^p58^ expression was significantly high in the stable expression group. CDK11^p58^ inhibited the expression of VEGF, CD31 and CD34 in breast tumors compared with the control group (Fig. 2a). Because VEGF is involved in promoting breast cancer angiogenesis, pseudocapillary formation in matrigel with HUVECs was first measured using the conditioned media of the two series of breast cancer cells. CDK11^p58^ stable expression and control breast cancer cells were cultured for 48 hours, then the conditioned medias were obtained. When plated in a thin layer of matrigel and stimulated with the conditioned medias, HUVECs were organized in a network of pseudocapillary tubes that invaded the gel (Fig. 2b). CDK11^p58^ treatment reduced the number of pseudocapillaries in terms of completed circles in MDA-MB-231 and T47D (Fig. 2c for quantification). These data suggest that CDK11^p58^ inhibited pseudocapillary formation in both MDA-MB231 and T47D.

Blood vessels of tumors were then examined. As shown in Fig. 2d and e, the density of blood vessels in MDA-MB231 tumors was attenuated significantly in tumors expressing high levels of CDK11^p58^ relative to control groups (Table 1). Both tumor size and the MVD (micro-vascular density) were inhibited by CDK11^p58^ in the MDA-MB231 group (Fig. 2d, e) and T47D groups (Additional file 1: Figure S1C). In addition, the vessel branches and nodes in the tumors were attenuated by CDK11^p58^. These data suggest that CDK11^p58^ inhibited breast tumor angiogenesis and proliferation *in vivo*.

**Table 1:** Detail information of vessels in tumors

	Branches	Nodes	Size (mm^3^)	MVD	Average OD (um)	Max OD (um)
T47D pBabe	18381	8000	1.5194	0.1465	17.6	138.22
T47D CDK11p58	7418	2943	0.4365	0.0984	13.43	73.01
231 pBabe	23560	10341	1.4792	0.891	17.71	123.31
231 CDK11p58	10166	3960	0.8135	0.0927	15.09	98.07

*OD*: Outside Diameter, *MVD*: Micro-vessel Density, *Size*: Tumor sample size, *Branches*: vessel branches, *Nodes*: vessel nodes

### CDK11^p58^ is associated with decreased angiogenesis in breast cancer patients

**Fig. 3:**
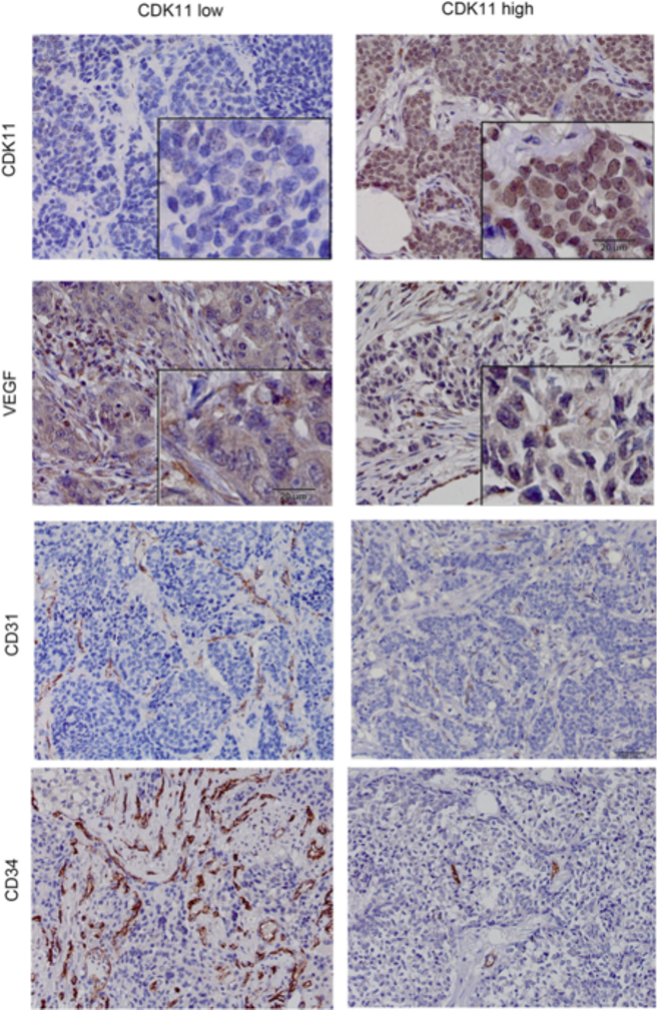
Association of CDK11^p58^ expression and VEGF expression in human breast cancer. Immunohistochemical staining for the expression of CDK11, VEGF, CD31, CD34 in human breast cancer tissues

To determine further whether CDK11^p58^ was involved in the regulation of angiogenesis in breast cancer, 32 breast cancer patient tumor tissues were used to examine the expression of CDK11^p58^ and angiogenesis related factors. VEGF, CD31, CD34 and CDK11p58 were examined by tissue array. CDK11^p58^ was expressed both in the nucleus and cell plasma. VEGF was expressed mainly in plasma. CD31 and CD34 were expressed specifically in vascular endothelial cells (Fig. 3). By IHC, we also observed high CDK11 expression in 18 cases and low expression in 14 cases. In the same patients’ tissues, high VEGF expression was observed in 15 cases and low expression was observed in 15 cases. The expression pattern of CDK11 was opposite to that of VEGF, CD31 and CD34 staining. The value of Chi-squared test for the correlation between CDK11 and VEGF was 10.041 and the P value was less than 0.01 (Table 2). The clinical data supported the negative association of CDK11^p58^ with VEGF and demonstrated CDK11^p58^ inhibited angiogenesis in breast cancer.

**Table 2:** Correlation of CDK11^p58^ and VEGF levels in breast cancer patients

	n	VEGF expression		×^2^	*p*
		Positive (%)	Negative (%)		
CDK11 positive	18	4 (12.5 %)	14 (43.7 %)	10.041	0.004
CDK11 negative	14	11 (34.4 %)	3 (9.4 %)		

### CDK11^p58^ inhibits angiogenesis by inhibition of the VEGF signaling pathway

**Fig. 4:**
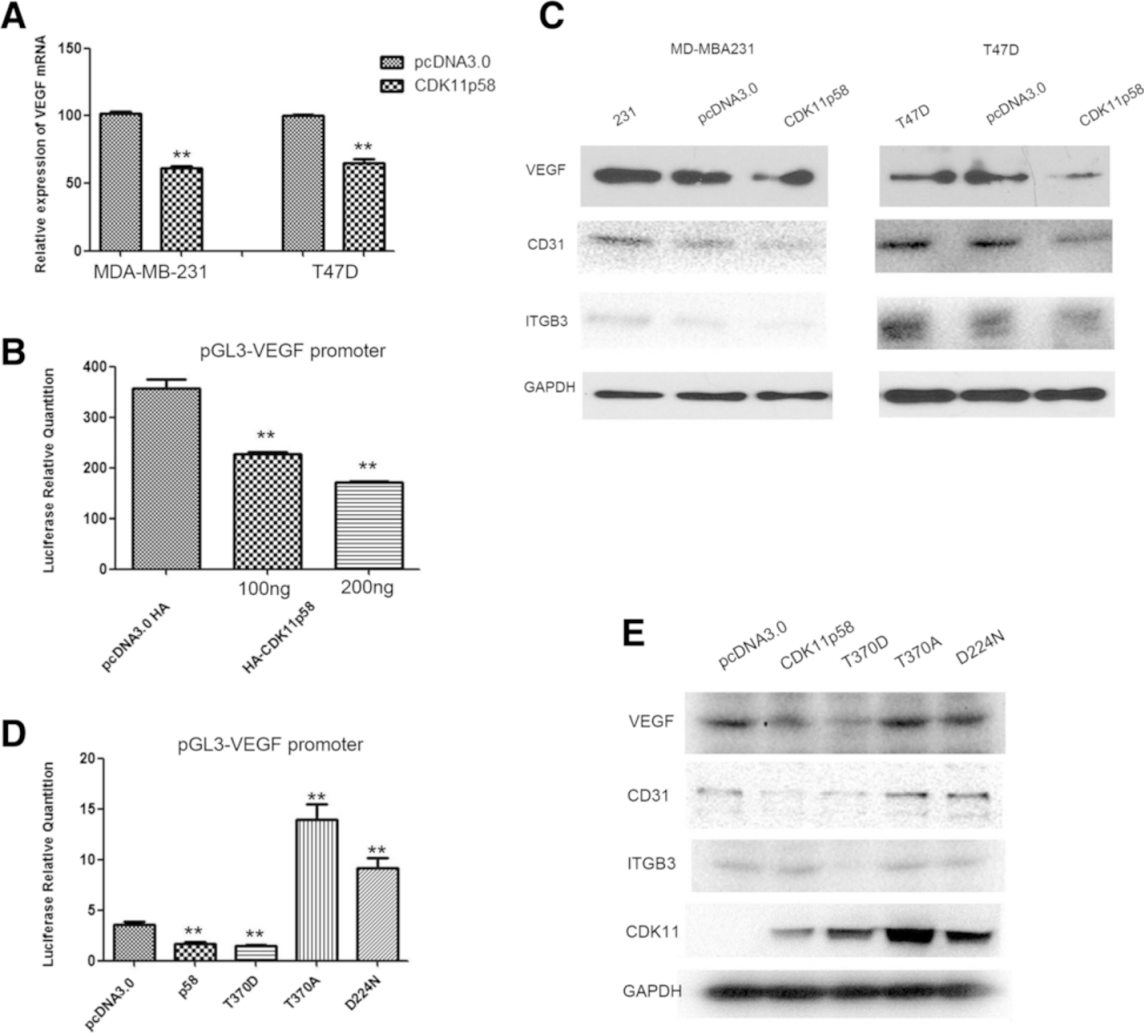
Regulation of VEGF signaling by CDK11^p58^. (**a**) qRT-PCR analysis of VEGF mRNA in breast cancer cells MDA-MB-231 and T47D. **P < 0.01 CDK11^p58^ group vs control group. (**b**) After transfection of CDK11^p58^ expression for 48 h, luciferase activity of VEGF promoter reporters was detected in 293 T cells. **P < 0.01 CDK11^p58^ vs control vector. (**c**) Western blot analysis of angiogenesis-related proteins by CDK11^p58^. (**d**) The luciferase activity of VEGF promoter reporters with CDK11^p58^ expression and CDK11^p58^ mutant expression in T47D cells. (**e**) Western blot analysis of angiogenesis related proteins by CDK11^p58^ and its mutations in MDA-MB231

To examine the regulation of VEGF by CDK11^p58^, VEGF mRNA was detected by qRT-PCR. VEGF mRNA was inhibited by CDK11^p58^ both in MDA-MB231 and in T47D (Fig. 4a). Promoter activity of VEGF assessed by Dual luciferase assay in 293 T demonstrated that CDK11^p58^ decreased the promoter activity of VEGF compared with the control in a dose dependent manner (Fig. 4b). In addition, CDK11^p58^ inhibited the protein expression of VEGF, CD31, and other angiogenesis-related protein integrin β3 (ITGB3) (Fig. 4c, the normalized quantification of immunoblotting data was shown in the Additional file 1: Figure S2A). CDK11^p58^ is a Ser/Thr kinase and whether inhibition was dependent on its kinase activity was then examined. T370A and D224N are CDK11^p58^ kinase dead mutants whereas T370D is a kinase-activated mutant as previously reported [12, 18]. T370D inhibited the activity similar to the wild type. However, T370A and D224N lost the inhibitory ability but promoted the activity of the VEGF promoter (Fig. 4d). These data suggest that CDK11^p58^ inhibited the promoter activity of VEGF in a kinase dependent manner. Western blotting also showed that CDK11^p58^ inhibited the expression of VEGF, CD31 and integrin β3 proteins in a kinase dependent manner in MDA-MB231 cells (Fig. 4e, the normalized quantification of immunoblotting data was shown in the Additional file 1: Figure S2B). Taken together, these data suggest that CDK11^p58^ inhibited angiogenesis through VEGF signaling in a kinase dependent way.

## Discussion

In this study, we focused on the critical role of CDK11^p58^ in breast cancer growth and angiogenesis, especially the regulation of VEGF by CDK11^p58^ and the dependence on its kinase activity. First, we determined that CDK11^p58^ inhibited the growth and formation of pseudocapillaries in breast cancer cells. Using a nude mouse model, CDK11p58 inhibited the growth and density of microvessels of the transplanted tumor. Second, by mice tumor tissues, we used IHC to determine a negative association of the expression of VEGF, CD31, and CD34 as well as MVD status with CDK11 expression. Similar results were observed in human breast cancer tissues. Then, we detected the regulation of VEGF by CDK11^p58^ both in 293 T cells and breast cancer cells. CDK11^p58^ inhibited the promoter activity of VEGF regulation at the transcriptional level and constantly inhibited angiogenesis-related protein expression in a kinase dependent manner.

Breast cancer is the most common female cancer and among the most frequent causes of cancer mortality in women worldwide [19, 20]. Cancer can spread through tissues, the lymph system and the blood [21]. Breast cancer is prone to travel through the blood vessels to other parts of the body, mainly to the brain, bone and lung [22–24]. Angiogenesis is a critical process in tumor growth and metastasis [25]. VEGF family members are involved in the regulation of angiogenesis. VEGF is the main component of this family and stimulates angiogenesis in health and disease by signaling through VEGF receptor-2 [3, 26]. Thus far, the VEGF-neutralizing antibody bevacizumab (Avastin) is used for metastatic colorectal, metastatic breast cancer and other metastatic cancers [25].

CDK11^p58^ is involved in a variety of important regulatory pathways in eukaryotic cells, including cell cycle control, apoptosis, neuronal physiology, differentiation and autophagy [10, 27–31]. It is a Ser/Thr kinase and most of its functions are dependent on its kinase activity [32]. In our previous study, we found that CDK11^p58^ repressed ERa transcription activity by promoting its ubiquitin-proteasome degradation in breast cancer [13].

In this study, we found that CDK11^p58^ inhibited the growth and angiogenesis not only in breast cancer cells but also in a nude mouse breast tumor model. This revealed that CDK11^p58^ might act as a tumor suppressor in breast cancer.

In the nude mouse cancer model and in the breast cancer patient samples assessed by IHC, we also found that CDK11^p58^ expression was negatively associated with angiogenesis related proteins VEGF, CD31 and CD34. Similar results were obtained in breast cancer cells. These data suggest that CDK11^p58^ might inhibit tumor proliferation and progression by an influence on angiogenesis.

As VEGF predominately regulates angiogenesis and several studies reported that targeting VEGF gene could inhibit the proliferation and induce the apoptosis of human breast cancer cells and in mice models [33–35], we speculated that CDK11^p58^ might inhibit angiogenesis through the regulation of VEGF. To confirm further the roles of CDK11^p58^ and VEGF, the mRNA levels of VEGF were examined at different levels of CDK11^p58^. We found that CDK11^p58^ inhibited VEGF mRNA and promoter activity of VEGF. These results indicated that CDK11^p58^ inhibited the angiogenesis of breast cancer by inhibiting the promoter activity of VEGF in a dose dependent manner. Based on our previous study, CDK11^p58^ could also induce the apoptosis of cancer cells through blocking the cells into the G2/M cell phase. So the mechanism involved in the growth and angiogenesis inhibition function of CDK11^p58^ should be complicated and not only dependent on the VEGF pathway. It needs further investigation.

As CDK11^p58^ is not a transcription factor, we speculated that VEGF promoter activity was indirectly influenced by CDK11^p58^. CDK11^p58^ might function as a co-repressor or regulate related transcription factors. The exact mechanism requires further investigation. In addition, CDK11^p58^ inhibited the protein expression of VEGF, CD31, and integrin β3. Several reports revealed that some breast cancer cells acquired CD31 expression [36]. CD31 expression mainly correlates with tumor cells spreading within the ductal system [37]. Additionally, CD31 can effluence the growth and differentiation of human breast cancer cells. Despite the expression level is relatively low in the breast cancer cells we investigated, CDK11^p58^ further inhibited its expression. Along with VEGF, it could further explain the inhibition effect of growth and angiogenesis by CDK11^p58^.

In our previous study, it showed that CDK11^p58^ could promote the ubiquitin–proteasome degradation of ER alpha [13]. In this study, the data showed that CDK11^p58^ inhibited the tumor growth and angiogenesis both in MDA-MB-231 ER-negative cells and in T47D ER-positive cells. Also, we found CDK11^p58^ inhibited VEGF promoter activity in MDA-MB-231, T47D and 293 T cells. So we speculated that it was ER independent. CDK11^p58^ inhibited the tumor growth and angiogenesis in an ER independent way.

CDK11^p58^is a Ser/Thr kinase and most of its functions are kinase-dependent. Thus, we hypothesized that VEGF inhibition was also CDK11^p58^ kinase dependent. Because we previously showed that Thr370 of CDK11^p58^ was responsible for CDK11^p58^ autophosphorylation, dimerization and kinase activity, mutant T370D and T370A were constructed. In addition, the mutant D224N was reported to be a kinase dead mutant. Indeed, the kinase constantly activated mutant T370D significantly inhibited the VEGF promoter activity compared with the kinase-dead mutant T370A and D224N. The same pattern was obtained at the protein level. These data suggest that the VEGF signaling pathway is inhibited by phosphorylation triggered by CDK11^p58^ and that CDK11^p58^ inhibits angiogenesis through VEGF signaling in a kinase dependent manner. CDK11^p58^ could function through phosphorylating some substrates to be involved in the regulation of VEGF transcription. Base on this result, we will further investigate its mechanism through finding CDK11^p58^ substrates by MS analysis.

## Conclusions

Taken together, our data show that CDK11^p58^ inhibits the growth and angiogenesis of breast cancer through inhibiting the regulation of VEGF signaling in a kinase activity dependent manner.

## Additional file

Additional file 1: Figure S1. (A) Colony formation of T47D cells stably transfected with CDK11^p58^ or pcDNA3.0. (B) Tumorigenesis after injection of T47D cells stably expressing CDK11^p58^ or control pBABE. Growth curve with CDK11^p58^ stable expression and controls was also shown below. (C) CDK11^p58^ inhibits the vascularization of tumors of T47D in mice. Figure S2. (A) Western blot analysis of angiogenesis-related proteins by CDK11^p58^. The normalized quantification of immunoblotting data from triplicate experiments were shown as below. (B) Western blot analysis of angiogenesis related proteins by CDK11^p58^ and its mutations. The normalized quantification of immunoblotting data from triplicate experiments were shown as below. **p* < 0.05; ***p* < 0.001. (DOC 1612 kb)

## Abbreviations

CDK11: Cyclin dependent kinase 11
CDC2L1: Cell Division Cycle 2-like 1
ER: Estrogen receptor
VEGF: Vascular endothelial growth factor
IHC: Immunohistochemistry
HUVEC: Human umbilical vein endothelial cells
CD31: Platelet endothelial cell adhesion molecule-1
MMP: Matrix metalloproteinase
ITGB3: integrin β3
