# Supplementary material for: Critical role of CDK11p58 in human breast cancer growth and angiogenesis
Source: BMC Cancer. 2015 Oct 15;15:701. doi: 10.1186/s12885-015-1698-7 (PMC4608324; doi:10.1186/s12885-015-1698-7)

**Additional file 1: Figure S1.** (A) Colony formation of T47D cells stably transfected with CDK11^p58^ or pcDNA3.0. (B) Tumorigenesis after injection of T47D cells stably expressing CDK11^p58^ or control pBABE. Growth curve with CDK11^p58^ stable expression and controls was also shown below. (C) CDK11^p58^ inhibits the vascularization of tumors of T47D in mice.


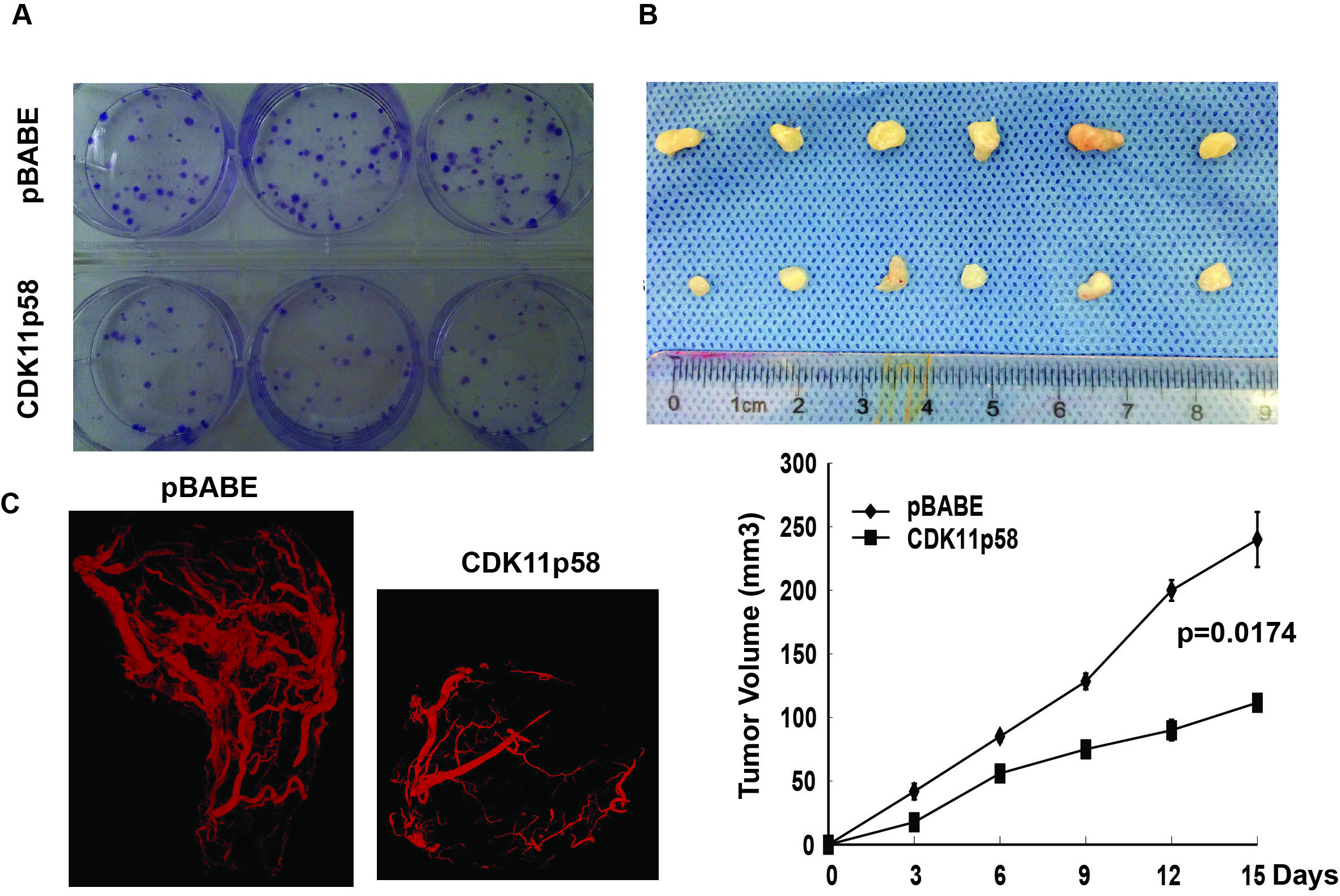


**Additional file 1: Figure S2.** (A) Western blot analysis of angiogenesis-related proteins by CDK11^p58^. The normalized quantification of immunoblotting data from triplicate experiments were shown as below. (B) Western blot analysis of angiogenesis related proteins by CDK11^p58^ and its mutations. The normalized quantification of immunoblotting data from triplicate experiments were shown as below. * p<0.05; **p<0.001.


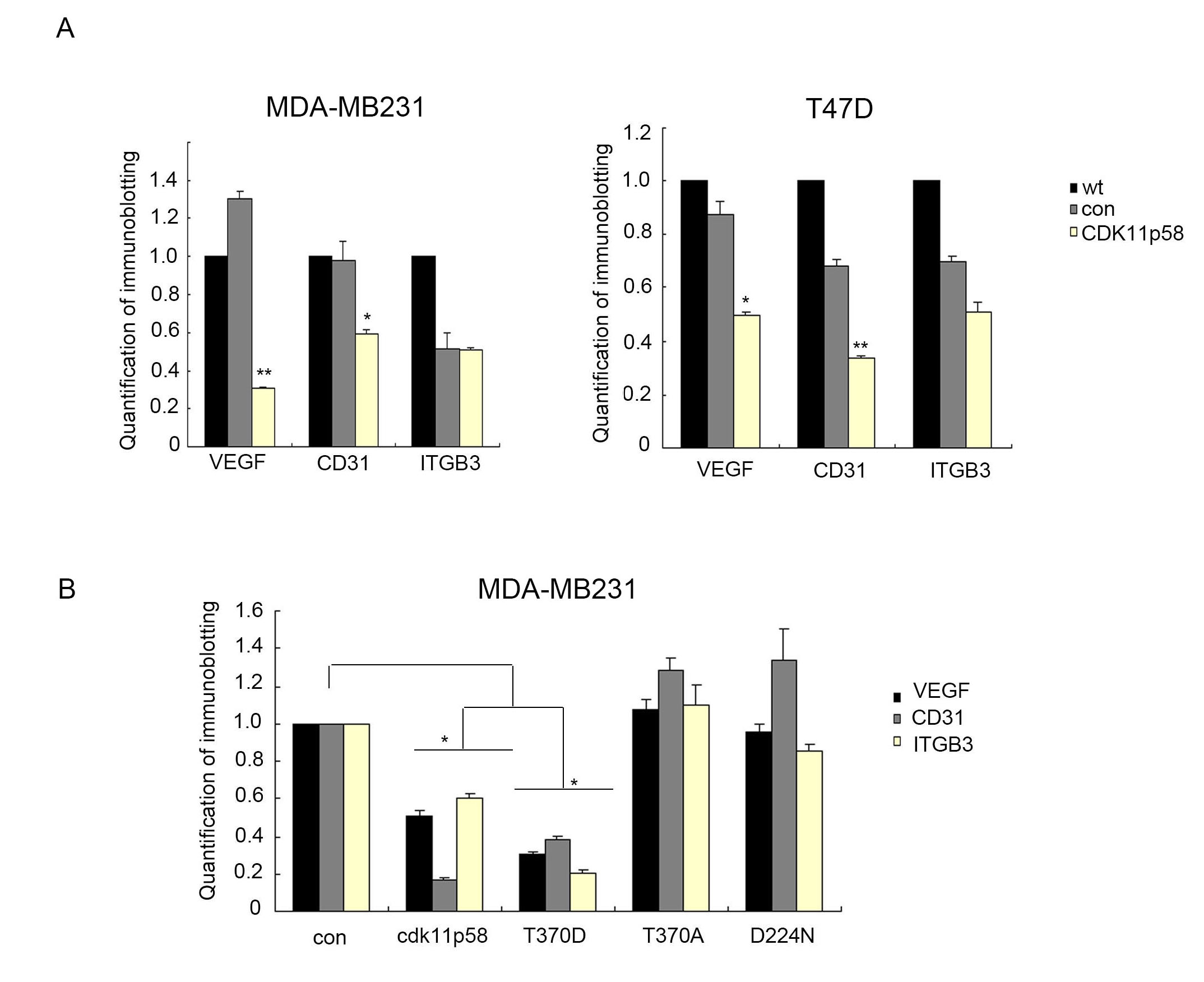

Supplement: Additional file 1: Figure S1. — (A) Colony formation of T47D cells stably transfected with CDK11p58 or pcDNA3.0. (B) Tumorigenesis after injection of T47D cells stably expressing CDK11p58 or control pBABE. Growth curve with CDK11p58 stable expression and controls was also shown below. (C) CDK11p58 inhibits the vascularization of tumors of T47D in mice. Figure S2. (A) Western blot analysis of angiogenesis-related proteins by CDK11p58. The normalized quantification of immunoblotting data from triplicate experiments were shown as below. (B) Western blot analysis of angiogenesis related proteins by CDK11p58 and its mutations. The normalized quantification of immunoblotting data from triplicate experiments were shown as below. *p < 0.05; **p < 0.001. (DOC 1612 kb) [file 12885_2015_1698_MOESM1_ESM.doc]
